# Familial Comorbidity of Bipolar Disorder and Multiple Sclerosis: Genetic Susceptibility, Coexistence or Causal Relationship?

**DOI:** 10.3233/BEN-2012-110198

**Published:** 2012-04-24

**Authors:** Mary H. Kosmidis, Vasilis P. Bozikas, Vaitsa Giannouli, Athanasios Karavatos, Konstantinos Fokas

**Affiliations:** ^1^School of PsychologyAristotle University of ThessalonikiThessalonikiGreece; ^2^1st Department of PsychiatrySchool of MedicineAristotle University of ThessalonikiThessalonikiGreece

**Keywords:** Multiple sclerosis, bipolar disorder, comorbidity, neuropsychology, neuroimaging, genetic

## Abstract

Our purpose in undertaking the present study was to examine the hypotheses proposed for explaining the frequent comorbidity of bipolar disorder and multiple sclerosis. One hypothesis posits that, when there is comorbidity, MS plays a causal role in psychiatric manifestations. Another suggests that both disorders have a common underlying physiological process that increases the likelihood of their co-occurrence. We examined two adult siblings with comorbidity and their relatives, including three generations of family members with psychiatric morbidity. We found an extensive multigenerational history of bipolar disorder in this family. This history would seem to support the hypothesis of a common underlying brain process (potentially genetically-based) to explain the comorbidity of BD and MS, but cannot clarify whether this comorbidity implies a relationship between the two disorders or merely reflects parallel processes of brain deterioration. We cannot, however, rule out the possibility of a subclinical MS-related process leading to the early manifestation of BD, with MS appearing much later in time, or even a third, undetermined factor, leading to familial comorbidity. Although we have insufficient information to support either hypothesis definitively, we present the familial cases as a springboard for a discussion of dilemmas related to teasing apart MS and BD comorbidity. Further observation of the clinical course of the younger family members, who have not yet shown any neurological signs, over the next few years may elucidate the current picture further.

## 1. Introduction

A plethora of studies have suggested an association between depressive spectrum disorders and multiple sclerosis (MS) [1–9]. Patients with MS are significantly more likely to present with histories of mania or manic psychosis [3,10,11], as well as with hallucinations or paranoid delusions (for a review, see [12]), than the inpatient psychiatric population as a whole. In fact, epidemiological studies have shown an association between MS and bipolar disorder (BD), with the prevalence of BD in MS patients being twice that of the general population [7].

Increased rates of comorbidity of BD and MS suggest a common neurological substrate for both diseases. Neuroimaging findings related to BD have shown structural abnormalities in the subcortical white matter and the temporal lobes of patients [13–16], while neuroimaging findings for MS have reported structural changes in subcortical white matter and the corpus callosum of diagnosed patients [17–19]. These findings suggest a similar subcortical white matter involvement in both diseases.

A recent review pertaining to the putative relationship between BD and MS presented evidence of parallel genetic and molecular processes, which may account for both disorders (as well as other related psychiatric disorders, namely, schizophrenia and major de-

ISSN 0953-4180/12/$27.50 © 2012 – IOS Press and the authors. All rights reserved pressive disorder) [20] The authors limited their presentation to the most consistent findings in the literature. Based on these, they concluded that in psychiatric disorders, genes expressed in oligodendrocytes (which myelinate axons to form white matter tracts), as well as those involved in energy metabolism and mitochondrial function, are downregulated, whereas genes involved in immune response and inflammation are upregulated. Similarly, the authors note, the extensive research on MS has shown a strong association among demyelination, inflammation, and energy metabolism. Thus, they attribute symptom differences between psychiatric disorders and MS to the extent of oligodendrocyte stress, wherein downregulation of oligodendrocyte genes results in abnormal myelination, and, thus, reduced neuronal functioning. Factors related to gene environment and expression, they speculate, may play a role in whether there is gradual and complete demyelination and subsequent cell death, as observed in MS, or demyelination that is partial and that occurs in particular brain regions, manifesting as psychiatric symptoms. Which process occurs may depend on (unspecified) factors in the genetic environment. By implication, the increased comorbidity of BD and MS may reflect a common molecular and neurological process, increasing the likelihood of their coexistence in the same individuals, regardless of any direct relationship between the two. If there is a relationship between the two disorders, however, its direction remains unclear based on current knowledge, as mitrochondrial and myelin abnormalities may be either the cause or the consequence of disease, and inflammation may be the cause or the consequence of the demyelination of cells under mitochondrial stress.

Neuropsychological investigations have also yielded common deficit patterns in BD and MS. More specifically, BD has been associated with impairments in learning and memory, attention, visuospatial skills, executive functions and verbal memory [21–27]. Similarly, MS patients show a decline in memory, attention, visuospatial, planning and organizational skills and abstraction, with additional deficits in language, cognitive flexibility and speed of information processing [28–36]. Of course, the degree and pattern of cognitive dysfunction in MS is highly related to the amount and location of white-matter disease within the cerebral hemispheres.

Particularly interesting is the appearance of comorbidity of BD and MS in families. In the current scientific literature there is only one published paper describing a mother and daughter who were diagnosed with MS and BD in the form of recurrent manic psychosis [37]. Clinical examination yielded a diagnosis of definite MS and BD for both patients. Magnetic resonance imaging (MRI) revealed multiple hypersignal lesions, especially in the right frontal lobe in both individuals, while genetic testing showed similar class I HLA alleles and identical class II HLA alleles. The authors concluded that the coincidence of neurological and psychiatric symptoms during MS relapses in these familial cases provided support for the hypothesis that MS played a causal role in the psychiatric symptoms manifested, as opposed to the hypothesis of the existence of a common genetic susceptibility, which might account for the comorbidity of BD and MS.

Elucidating the mechanism contributing to the frequent comorbidity of BD and MS may be important for two reasons. It may lead to a better understanding of the pathophysiology underlying each of these disorders. Additionally, it may have implications for the optimal pharmacological treatment of psychiatric manifestation in light of neurological symptoms. Therefore, in the present study we explored the comorbidity of BD and MS in two first-degree relatives (adult siblings), but also the existence of psychiatric morbidity in several other family members. We present their clinical status at initial assessment and their clinical course over the past 10 years along with information regarding their parents (one healthy, the other with a history of BD) and a description of the psychiatric diagnoses of the two adult daughters of one of the siblings. In describing these cases, we sought to discern patterns that might support or refute either of the potential hypotheses regarding the comorbidity of BD and MS. We hypothesized that a causal role of MS in the development of BD would be likely, if the MS either preceded the BD or if the BD symptoms manifested during MS relapse, as reported previously [37]. In contrast, we hypothesized that a common pathophysiology for MS and BD would be likely (whether they are related directly or independent of each other), if BD preceded the onset of MS and if the relapses of the two disorders were temporally independent of each other. Finally, we reasoned that any familial and/or multigenerational morbidity or comorbidity would support the possibility that there is a genetic susceptibility for the comorbidity of BD and MS.

## 2. Patients

We began to follow the family reported in the present study in 2001. This family has had three generations of BD. Two of the patients (A and B – adult siblings) had a long history of bipolar disorder with a recent diagnosis of MS. Their father (deceased) and patient A's eldest daughter (patient C; 25 years old at the time of testing) had been hospitalized for manic episodes, while patient A's youngest daughter, patient D (24 years old at the time), had no psychiatric history then (she was hospitalized several years later, at age 29, for a brief psychotic episode). Four of the family members discussed in this report [patients A and B, their mother (that is, the grandmother of the family) and patient C, the eldest daughter of patient A] were examined regarding BD and MS comorbidity and HLA typing (reported in [37]). The three affected members and the non-affected grandmother shared the same class I and II, HLA-A2, B18, CW8, DR2, DQ1 haplotype, which is considered to be associated with MS. This finding could also indicate a possible genetic locus on chromosome 6 (close to the HLA region) for BD [38].

### 2.1. Grandmother

On initial assessment of the family in 2001, the grandmother's medical history, neurological examination and auditory (AEPs) and visual evoked potentials (VEPs) were normal. Her brain MRI was also normal, with the exception of mild multi-infarct lesions consistent with her age. She had no other testing. Since that time, she has had at least one stroke.

### 2.2. Patient A

Patient A, a right-handed divorced woman with a six-year education (which was the minimum compulsory education at the time), was 43 years old at initial assessment (in 2001). She had received a diagnosis of Bipolar Disorder I (BD I; treated with mood stabilizers) 17 years previously and had a history of multiple hospitalizations for six manic episodes in the past. The patient initially presented to our psychiatric clinic with symptoms of muscular weakness and manic exacerbation (euphoric mood, grandiose ideas, flight of ideas, distractibility, disinhibition, and psychomotor agitation). Upon admission, she presented with left lower extremity weakness and unstable gait, symptoms that she reported had appeared one week earlier. A neurological examination revealed hyperreflexia of the left extremities, Babinski reflex on the left, markedly decreased motor strength and moderately decreased touch and pain sense at $T_{12} - O_1$ level on the right side. She had experienced episodes of mild blurred vision and

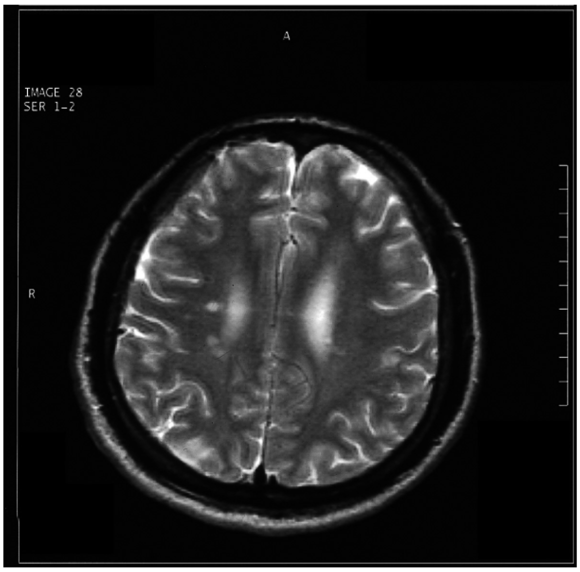

Fig. 1. MRI of patient A showing demylienation in periventricular and subcortical white matter and lesions in the semiovale bilaterally, right pons and right cerebral cortex.

diplopia four years previously, but as these episodes had lasted only a few weeks she had not mentioned them to her psychiatrist at the time. This neurological examination yielded a diagnosis of definite MS [38] (after having ruled out potential differential diagnoses in a comprehensive neurological work-up) with mild symptoms (EDSS = 3).

Neurophysiological findings for this patient suggested mild abnormalities, while neuroimaging showed definite brain lesions. More specifically, AEPs revealed a vague right response and a normal left response, while VEPs revealed a normal right response and an undetectable left response. Somatosensory evoked potentials (SEPs) were normal bilaterally. Computerized tomography (CT) was normal, but MRI portrayed diffuse, old and new lesions consistent with demyelination in periventricular and subcortical white matter and lesions in the semiovale bilaterally, right pons and right cerebral cortex (Fig. 1).

Patient A participated in an extensive neuropsychological assessment in 2001, approximately one year after her diagnosis of MS. Both her disorders were in remission at the time of the evaluation. Overall, patient A's performance on the neuropsychological battery was poor (Table 1). Her scores on measures of attention were all in the impaired range. Immediate and delayed recall scores were poor for both verbal and visual information. She was able to learn a list of words with repetition, but unable to recall them after the learning

Table 1
Neuropsychological test performance of patients A, B and C

| Tests | A | Impairment | B | Impairment | C | Impairment |
|---|---|---|---|---|---|---|
| *Attention* | | | | | | |
| Trail Making Test Part A (sec) | 145 | Severe | 130 | Severe | | |
| Digit Span (WAIS-III)* (ss) | 5 | Moderate | 4 | Moderate | | |
| Digit Symbol (WAIS-III) | 2 | Severe | 3 | Severe | 4 | Severe |
| Symbol Copy (WAIS-III) | 1st | Severe | 1st | Severe | 30th | Severe |
| Stroop Word | 76 | Mild | 81 | Mild | 88 | Severe** |
| Color | 49 | Moderate | 52 | Mild | 60 | Severe** |
| Color/Word | 18 | Moderate | 20 | Mild | Declined | – |
| Spatial Span (WMS-III) | 3 | Mod-Severe | 3 | Mod-Severe | | |
| Arithmetic (BAT) (%) | 36 | Moderate | 21 | Moderate | | |
| *Memory* | | | | | | |
| Rey-Osterrieth Complex Figure Immediate Recall | 4 | Severe | 3 | Severe | | |
| Delayed Recall | 0 | Severe | 4.5 | Severe | | |
| Recognition | Declined | – | 19 | Moderate | Declined | – |
| Hebb's Recurring Digits | 0 | Severe | 0 | Severe | | |
| Word List Learning 5th trial | 10 | Mild | 5 | Moderate | | |
| Free short recall | 4 | Moderate | 5 | Moderate | | |
| Cued short recall | 12 | Mild | 7 | Moderate | | |
| *Language* | | | | | | |
| Semantic categorization (%) | 98 | Normal | 70 | Mild | | |
| Semantic Fluency (total # words) | 19 | Mod-Severe | 34 | Moderate | | |
| Phonological Fluency (total # words) | 9 | Mod-Severe | 0 | Mod-severe | | |
| Bilingual Aphasia Test Word/Pseudoword Recognition (%) | 53 | Mild | 25 | Mod-severe | | |
| Comprehension (%) | 100 | Normal | 80 | Mild | | |
| *Visuoperceptual skills* | | | | | | |
| Benton's Face Recognition | 39 | Severe | 28 | Severe | 34 | Severe |
| Bell Test (%) | 97 | Normal | 94 | Normal | | |
| Picture Completion (WAIS-III) | 1 | Severe | Declined | – | 1 | Severe |
| Picture Arrangement (WAIS-III) | 0 | Severe | 5 | Mod-severe | | |
| Rey-Osterrieth Complex Figure Copy | 6.5 | Severe | 22 | Moderate | | |
| Hooper Visual Organization Test | 10 | Severe | 8 | Severe | 11 | Moderate |
| *Executive Functions* | | | | | | |
| Tinkertoy Test (%) | 25 | Mod-severe | 75 | Normal | | |
| Trail Making Test Part B (sec) | 325 | Severe | Declined | – | | |
| Wisconsin Card Sorting Test Perseverative errors (% ) | 87 | Severe | Declined | – | 25 | Moderate |
| *Motor Skills* | | | | | | |
| Grooved Pegboard Dominant hand (sec) | 370 | Severe | 205 | Severe | | |
| Non-dominant hand (sec) | 345 | Severe | 144 | Moderate | | |
| *Affect Perception* | | | | | | |
| Prosody (% correct) | 36 | Moderate | 33 | Moderate | 60 | Normal |
| *Interhemispheric Transfer* | | | | | | |
| Dichotic Listening Test (ratio) | 0.08 | Symmetrical | −0.04 | Symmetrical | −0.083 | Symmetrical |

*WAIS-III = Wechsler Adult Intelligence Scale-III; WMS-III = Wechsler Memory Scale-III; BAT = Bilingual Aphasia Test.
**The comparison was made with the same age group and 10–12 years (formal high school) education, because of the lack of a normative sample of adults in the same age range with fewer than 9 years of education (compulsory education in Greece).

trials. Tests of verbal processing showed good categorization skills with the use of semantic information and comprehension of grammatical structures. Although she could repeat a list of real words and pseudo-words flawlessly, she mistakenly judged many of the latter to be real words. Her verbal fluency scores were low for both semantic and phonological categories; her output was very poor, with two perseverations and no rule infractions.

The patient's visuoperceptual skills were also quite poor. Her performance on a face recognition task was in the borderline range and the copy of a complex figure was below the 10th percentile, since mental rotation and integration of parts were severely impaired as she focused on individual components and did not perceive the whole. On a picture arrangement test, her score was in the very low range. Measures sensitive to executive dysfunction were also poor. Her time to completion of Trail Making-Part B was very long, although she made no errors. On a less structured test, she was unable to construct a nameable object that was functional. Card sorting was discontinued before completion due to her difficulties performing this task and motor processing was slow. She showed a mild right ear advantage on a dichotic listening task. Finally, her perception of prosody was poor, as she responded correctly to only 50% of the items; she appeared to perseverate on the emotion of happiness, which was presented in the first trial.

Since 2004, patient A has had 12 hospitalizations increasing in duration each time, the most recent one lasting five months, before her condition had stabilized enough to justify discharge. She is currently 54 years of age. During her last hospitalization, her clinical profile included euphoria, disinhibition, irritability, emotional lability (sudden onset crying bursts that were superficial and subsided equally suddenly) and occasional depressive symptomatology. Cognitive deterioration over the years in memory, language, communication and orientation suggesting a dementing process is evident, perhaps due to her MS. She is not currently capable of participating in a formal neuropsychological assessment, however, given her almost lethargic state. For example, she gets lost in the clinic (cannot find her room) and occasionally does not recognize one of her attending psychiatrics (VPB), whom she has known for 15 years, while her speech content is often incoherent. When not hospitalized, she lives with her elderly mother and brother with very poor daily functioning.

### 2.3. Patient B

Patient B, the brother of patient A, was 40 years old during the assessment (in 2001) with a 20-year history of BD I and many hospitalizations in psychiatric clinics. He was not hospitalized at the time of the examination, though he was in a hypomanic state and was taking mood stabilizers. He is right-handed and has completed elementary school (minimum compulsory education). Patient B reported an episode of lateral blindness three years prior to the initial evaluation. Neurological examination revealed mild pyramidal signs on the left side, while neurophysiological studies showed

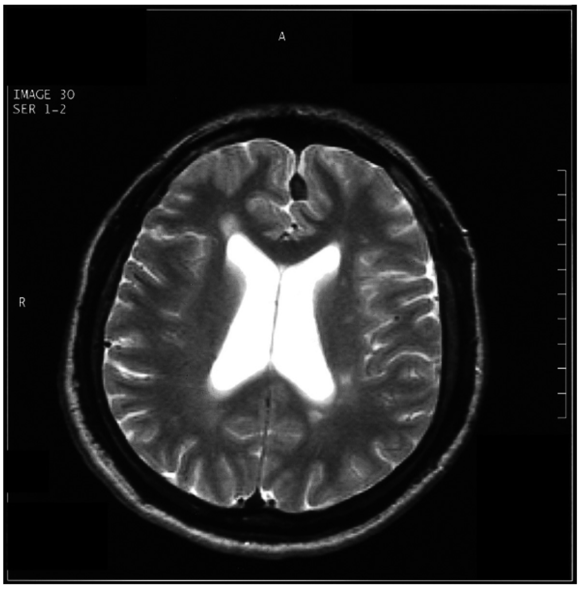

Fig. 2. MRI of patient B showing periventricular and subcortical white matter lesions with additional bilateral demylienation of the centrum semiovale and the corpus callosum.

mild abnormalities. More specifically, AEPs showed a vague response bilaterally, VEPs elicited an attenuated right response, with no response on the left, while his SEPs were characterized as borderline. A brain MRI depicted diffuse, old periventricular and subcortical white matter lesions with additional bilateral lesions of the centrum semiovale and of the corpus callosum consistent with demyelination (Fig. 2). He was given a diagnosis of definite MS [39] (after having ruled out potential differential diagnoses in a comprehensive neurological work-up) with mild symptoms (EDSS = 3), but was not taking any medication for it.

Patient B's performance on the same extensive neuropsychological battery was also quite poor. The majority of his scores on measures of attention indicated severe difficulties. These difficulties were similar to his sister's, with the only exception of a better (but still lower than normal) performance on the Stroop test. His performance on memory tests was severely impaired on both immediate and delayed recall for visual information, while his recognition performance was in the range of moderate impairment. Similarly, his memory for verbal information showed a moderate impairment as he was able to learn only a few words from a list even with repetition, and with equally impaired delayed recall. Tests of verbal processing showed mildly diminished categorization skills. His verbal fluency was low for both semantic and phonological categories.

The patient's visuoperceptual skills were also quite poor, as his performance on a face recognition task was in the severely impaired range. His copy of a complex figure was moderately impaired. Visual sequencing, as measured by a picture arrangement test, was in the moderately to severely impaired range. Measures sensitive to executive dysfunction yielded poor performance. His very slow performance on Trail Making-Part B led to discontinuation of the test, but on a less structured test, he was able to construct a nameable object that was functional. Card sorting was also discontinued due to his difficulties, while motor responding was slow. Finally, his score on an emotional prosody perception test indicated moderate impairment.

Over the past 10 years, patient B has not been hospitalized again to our knowledge, but has shown poor compliance with his medication and follow-up medical appointments. Therefore, information regarding his medical condition and course is sparse. What is known, however, is that he demonstrates poor functioning and thought disorder when non-compliant with his medication regimen, yet he presents considerable improvement when taking his medication appropriately, and is polite and cooperative.

### 2.4. *Patient C*

Patient C, the eldest daughter of patient A, was a single, right-handed 25-year old at the time of her assessment (in 2001), who also had only an elementary school education (6 years, at a time when compulsory education had increased to 9 years), with a diagnosis of BD I. Neurological examination and auditory (AEPs) and visual evoked potentials (VEPs) were normal; she declined the neuroimaging examination and terminated the neuropsychological assessment prematurely, refusing to complete the assessment at another date (Table 1). Her performance on the few tests she completed indicated poor cognitive functioning overall. More specifically, her visuospatial performance was in the moderately to severely impaired range. Performance on a card sorting task was moderately impaired and on attention severely impaired. In contrast to her mother and uncle, patient C's prosody perception was normal. She is currently 35 years of age and has not shown any signs of MS. Her medication regimen includes Abilify 30 mg. On a recent attempt to follow up with a repeated neuropsychological assessment, she declined. Therefore, no further information is available regarding her cognitive condition.

### 2.5. *Patient D*

Patient D's medical history, neurological examination and auditory (AEPs) and visual evoked potentials (VEPs) were normal at initial assessment of the family (in 2001), nor had she presented any psychiatric symptoms yet. She, too, has a low level of education (6 years). Both sisters (Patients C and D) grew up in foster institutions due to their mother's illness and their father's absence (he abandoned the family). At that time, she had declined neuropsychological and neuroimaging examinations. In 2006, at age 29 years, she was admitted to our clinic for an acute psychotic episode, received a diagnosis of brief psychotic disorder and was prescribed Amilsupride (800mg), which she took for two years. Again she declined neuropsychological and neuroimaging evaluations. She is now 33 years old and has presented neither psychotic nor MS related symptoms.

## 3. Discussion

We investigated the hypotheses regarding the frequently observed comorbidity of BD and MS by examining a family with three generations of psychiatric disorder, some members of which also had comorbid BD and MS. At least one previous report of familial comorbidity supported the hypothesis that MS-related processes play a causal role in the psychiatric manifestation of the patients described [37]. This was based on the temporal relationship of the two disorders, wherein psychiatric symptoms occurred during MS relapses. In contrast, the association between BD and MS in the present study was not definitive, but, instead, raised several possibilities. Although BD was diagnosed more than a decade previously to the MS diagnosis in both of our patients with comorbidity, we cannot rule out the possibility, based on the information available to us from the patients, that a subclinical MS-related process led to the initial presentation of BD, while MS was identified many years later. Alternatively, our patients' comorbidity might also suggest the existence of an underlying common pathophysiology for the two disorders, which may account for their co-occurrence. Whether these two disorders are related to each other or merely share common physiological processes leading to their co-occurrence in the same individuals, however, cannot be answered by the present family study.

Extensive neuropsychological assessment and neuroimaging of some of the family members in the present investigation were consistent with the subcortical patterns reported in the literature for both MS and BD. More specifically, the neuropsychological profiles of the three patients tested (patients A, B and C) were very similar: moderate to severe impairment in attention, encoding and retrieval, visuospatial perception, verbal fluency, motor skills, arithmetic, and executive functioning, with relatively intact semantic processing, retention of information, visual scanning, verbal comprehension, and abstract reasoning. We should note here, however, that the patients' poor cognitive functioning may also reflect their low level of education, which, in turn may reflect a low intellectual level or early cognitive deterioration - perhaps a precursor of their impending psychiatric and neurological disorders – leading to their premature discontinuance of school. Leaving school after completing the elementary level, however, was not atypical in small villages like the one in which the family lived; in fact, Patient A's daughters were raised in foster institutions due to their mother's illness and their father's absence (he abandoned them and raised a new family). Thus, the daughters' premature dropping out of school may also reflect limited resources and potential neglect by those in charge of their care. Similarly, MRIs for patients A and B revealed diffuse old and new periventricular and subcortical white matter lesions with additional demyelination of the centrum semiovale bilaterally, typical of both disorders. We also found that the three family members with BD (patients A, B, and C) as well as the non-affected grandmother, also shared the same genetic profile, which is thought to be associated with MS, suggesting that genes very close to the HLA region of chromosome 6 may be, at least in part, related to the manifestation of affective disorder [38].

Given the available information, we cannot explain sufficiently why some of the family members, but not others, developed both BD and MS. The fact that the two family members with comorbidity were of the same generation may suggest the involvement of genetic and/or environmental factors predisposing them to both disorders. These factors may have not been present in the grandfather or the daughters, sparing them of the MS.

One limitation to the generalizability of the present findings and our conclusions relates to the case study methodology used. Unfortunately, we did not have access to a series of multiplex families with MS in order to estimate the actual prevalence of BD in such families. Therefore, we cannot rule out the possibility that a common underlying brain process may merely lead to the development of each of these disorders without any other association between the two. Also unclear are the factors contributing to the severity of the demyelination observed in Patient A, as evidenced in her extreme cognitive decline in middle age. While this may reflect the severity of her current demyelination, one might speculate that her dropping out of school prematurely was due to an early onset of cognitive deterioration – a precursor, perhaps, of her impending psychiatric and neurological disorders. Although considered rare, several cases of patients with dementia (predominately of a subcortical nature) due to MS have been reported in the literature [40,41]. Patient A may be one of these rare cases. The possible scenarios explaining her dementia include the following: (a) early onset dementia that is autosomal dominant; (b) random comorbidity; (c) early onset dementia due to a genetic predisposition for all three diagnoses, namely, dementia, MS and BD; (d) early onset dementia due to MS, but more severe than usual. There is no known family history of dementia, thus her dementia does not appear to reflect hereditary factors.

The extensive multigenerational history of BD in the family described in the present report would seem to support the hypothesis of a common underlying brain process (potentially genetically-based) to explain the comorbidity of BD and MS, but cannot clarify whether this comorbidity implies a relationship between the two disorders or merely reflects parallel processes of brain deterioration. Thus, the BD may have resulted from biological mechanisms related to the MS or from those putative mechanisms which the two disorders have in common. We cannot, however, rule out the possibility of a subclinical MS-related process leading to the early manifestation of BD, with MS appearing much later in time, or even a third, undetermined factor, leading to familial comorbidity. Finally, the fact that BD is known to have a stronger hereditary component than MS may be viewed as supporting the possibility of two pathophysiological processes affecting some family members independently of each other. Further observation of the daughters' course over the next 10 years may elucidate the current picture.

## Acknowledgment

We would like to acknowledge the contribution of Drs. Nikolaos Scarmeas and Christina H. Vlahou.
